# Impact of mother’s education on full immunization of children aged 12–23 months in Eritrea: population and health survey 2010 data analysis

**DOI:** 10.1186/s12889-020-8281-0

**Published:** 2020-02-22

**Authors:** Fitsum Kibreab, Sonia Lewycka, Andebrhan Tewelde

**Affiliations:** 1 Ministry of Health of Eritrea/Health Research and Resources Centre Division, Asmara, Eritrea; 2 Oxford University Clinical Research Unit, Oxford, UK; 3 Ministry of Health of Eritrea/Monitoring and Evaluation Division, Asmara, Eritrea

**Keywords:** Full immunization, Coverage, Maternal education, Child 12–23 months, Eritrea

## Abstract

**Background:**

Although vaccination coverage in Eritrea has improved in recent years, some children are still missing out, and it’s important to identify risk factors for lower coverage in order to target campaigns and interventions. The objective of this study was to assess: (1) the impact of maternal education on full immunization of children aged 12–23 months, and (2) whether the association was confounded or modified by other factors.

**Methods:**

This study was a secondary data analysis of the Eritrean Population and Health Survey 2010 (EPHS 2010). In this analysis 1323 mothers of children aged 12–23 months were included. The outcome of the study was full immunization, defined as receiving all the WHO recommended basic vaccines: one dose of Bacillus Calmette-Gué rin (BCG), three doses of diphtheria-pertussis-tetanus(DPT), three doses of polio, and one dose of measles vaccine. The primary exposure was maternal education. Data on immunization coverage came from vaccination cards and from mothers’ or caretakers’ verbal reports. Bivariate and multivariable logistic regression analyses were performed.

**Result:**

Full vaccination coverage among children aged 12–23 months was 83%. Most children received BCG (95%), DPT1 (97%), DPT2 (96%), DPT3 (93%), polio1 (97%), polio2 (97%), polio3 (91%) and measles (92%). In unadjusted analyses, children of mothers with primary (OR = 2.75, 95% CI 1.74–4.37), and middle or above (OR = 3.16, 95% CI 2.09–4.78) education were more likely to be fully immunised. However, after adjusting for wealth, region, ANC visit, and vaccination card ownership, only the effect for primary education remained significant (OR = 2.34, 95% CI 1.30–4.21).

**Conclusion:**

The result of this study suggested that children of mothers who attained primary level were more likely to be fully vaccinated than children of mothers with no education. The association was influenced by wealth index of household, mothers ANC visit, region, and possession of vaccination card. The Expanded Program on Immunization of the Ministry of Health should target strategies to enhance full immunization among children of mothers with no education.

## Background

Immunization is a proven tool for controlling and even eradicating infectious disease [1]. Every year immunisation prevents 2 to 3 million infant deaths globally [2]. Though child immunization has been very successful, vaccine preventable diseases remains a major health problem among children in low and middle income countries [3]. WHO estimated that vaccine preventable diseases are still responsible for 1.5 million child deaths each year due to inadequate vaccination coverage: partially vaccinated, completely unvaccinated, or received vaccine later than scheduled [4]. The Global Immunisation Vision and Strategy, 2006–2015 set the target for at least 90% national vaccination coverage; however, global vaccination coverage for the proportion of children who received the eight WHO recommended basic vaccines (one dose of Bacillus Calmette-Guérin (BCG), three doses of Diptheria-Pertussis-Tetanus (DPT), three doses of Oral Polio Vaccine (OPV), and one dose of Measles Containing Vaccine (MCV)), has stalled at 86%. The Global Vaccine Action Plan (GVAP) 2016–2020 strategy, is more directly focused on reaching all children, regardless of whether their parents are rich or poor, whether they live in an urban slum or a wealthy neighbourhood, or whether their mothers are educated or not. This framework was set to help countries to achieve universal child immunization by 2020. Despite all these efforts almost 19.9 million infants were not given routine immunization in 2017 [4].

The Eritrea Expanded Program on Immunization (EPI) is led by the EPI unit under the division of Family and Community Health of the Ministry of Health (MOH). It was launched in 1980 when Eritrea was under the colonialism of Ethiopia with the following vaccines: BCG, DPT, OPV, and MCV [5]. The EPI program provides routine immunization activities in static health facilities and outreach sites and conducts national immunization days to boost the coverage [5]. According to the Eritrea Population and Health Survey (EPHS) 2010, 75.5% of children had received all the recommended vaccines by the age of 12 months, and 83% of children received full vaccination by the age of 23 months. Only 2.1% of children had received none of the recommended vaccines by the age of 12 months, and 1.8% by the age of 23 months [6]. According to the GAVI estimate, the national DPT3 coverage estimate for 2016 was 93%, but there was variability between districts, with 48% of districts achieving more than 80% DPT3 coverage, and 12% achieving less than 50% DPT3 coverage, however, it was not stated which districts were doing well and which district were doing poor [7].

It has been hypothesized that children of mothers who are educated are more likely to complete their vaccination because maternal education helps a mother to follow health seeking behaviours that enhance child vaccination [8]. To assess the determinants of child immunization in general and the association of maternal education and immunization coverage in particular, studies have been carried out in different settings and different populations. A survey conducted in Eritrea in 2017 among children aged 24–35 months to assess the national EPI coverage, found that 92% of children of mothers with middle or higher education were fully immunized compared with only 78% of children of mothers with no education [6]. other studies conducted in Nigeria and Ethiopia found that children of educated mothers were more likely to be fully immunized than children of uneducated mothers [9–11]. Conversely, a study conducted in Uganda in 2010, found that infants whose mothers had a secondary education were at least 50% less likely to miss scheduled vaccinations compared to those whose mothers had only primary education [12].

The association, mothers’ education and immunization coverage, controlling the effect of other variables on this association, in children aged 12–23 in Eritrea has not been investigated. This research, therefore, aimed to assess the impact of maternal education on full immunization in children aged 12–23 months in Eritrea, and to examine whether or not the association was confounded or modified by other factors.

## Methods

### Study setting

The Eritrean Population and Health survey (EPHS) 2010 was conducted in all regions of Eritrea known as Zoba: Anseba, Debub, Debubawi Keih Bahri (DKB), Gash-Barka, Maekel and Semenawi Keih Bahri (SKB). Besides, it included both urban and rural areas.

### Data source

This study was a secondary data analysis of the EPHS 2010 survey. The EPHS2010 survey was the third of its kind in a series that started in 1995 and then in 2002. Data were collected from January to July 2010. The EPHS 2010 data included a women’s questionnaire that measured background characteristics of the woman, and immunization and health status of her children under five years old, as well as child-specific information for all births in the past five years. There was also a household questionnaire that included socio-economic characteristics of the households and their usual members as well as anthropometric data (weight and height) of all children under five. The survey collected information on vaccination coverage in two ways: from vaccination cards shown to the interviewers, and from mothers’ or caretakers’ verbal reports. If the card was available, the interviewer recorded all the vaccination information from the card; but if there was not a vaccination card or vaccination information was not on the card the mother/care giver was asked to remember it.

### Sampling and sample size

The sample for the EPHS 2010 survey was selected in two stages. In the first stage 900 clusters were selected: 525 from rural areas and 375 from urban areas. In the second stage 20 households were selected using linear systematic sampling from each cluster, which gave a sample of 35,000 households in total. All women aged 15–49 who were either permanent residents of the households or visitors present in the household on the night before the survey were eligible to be interviewed. A total of 10,805 eligible women were found in these household, but 10,243 (94.8%) women were interviewed. There were 7013 children aged 0–59 months in the survey. In this analysis 1323 mothers with a child aged 12–23 months were considered and one child per woman was included. According to the WHO reference manual on evaluation of routing vaccination coverage, the target populations are defined in 12-month group and it has also specified the eligibility criteria for the population that would be included in the survey [13]. Besides, it recommended that the evaluation should target population of children aged 12–23 months if the final recommended basic vaccine is given at the age of nine months. In Eritrea the last primary vaccine (measles), one of these basic vaccines defined in this study, is given at the age of nine months; hence, this study had considered children aged 12–23 months to assess the relationship between maternal education and immunization. The sample size of this analysis, since it was a secondary data, was predetermined; it was fixed (*n* = 1323). However, to assess whether or not the data analysis would have enough power, a power calculation was done. The power was calculated based on the proportion of full immunization coverage status and maternal education level with 5% level of significance, a design effect of 1.23. The proportion of fully immunized children (p) with their sample size(n) of the EPHS 2010 was 74.6% (587), 88.9% (295), 89.5%(228), and 91.0%(213) for children of mothers with no education, primary, middle school, and secondary or above level respectively. The power estimation was done using the sampsi command of stata, sampsi p1 p2, n1 n2 alpha (0.05). So based on these coverage and sample sizes of mother’s educational level, the analysis had a power of greater than 90%.

### Variables

#### Outcome variable

The outcome variable was immunization status and it was categorized as “fully immunized”, and “not fully immunized”. Full immunization was defined as a child aged 12–23 months who had received all the WHO recommended basic vaccines – one dose of BCG, three doses of DPT, three doses of polio, and one dose of measles vaccine. A child who missed at least one dose of those recommended vaccines was considered as not fully immunized. The outcome variable, full immunization was coded as “Yes” if the child was fully immunized and “No” if the child was not fully immunized.

#### Exposure variables

The primary exposure variable was mothers’ education categorized as no education, primary, and middle or above. The socio-economic and demographic factors considered in this study were selected and coded based on the literature review. The variables considered in this analysis were maternal characteristics (maternal age, marital status, religion, occupation, exposure to media, antenatal visit, postnatal check-up, distance to health facility), child characteristics (sex of child, birth order, place of delivery, ownership of vaccination card), fathers’ characteristics (educational level, occupation) and household characteristics (place of residence, region, wealth index). In this study a mother referred to either the mother of the child or care giver; similarly, a father referred to either the father of the child or partner of the child’s mother.

Mother’s occupation, measured as paid work in the last 12 months’ prior the survey, was coded into four categories and then recoded into two categories: “currently working”- those who ever worked in the past 12 months; “currently not working “- those who never worked in the past 12 months prior the data collection. Wealth index was measured based on ownership of household assets such as car, bicycle, television radio as well as dwelling facilities such as drinking water, sanitation facilities and type of flooring material. Principal components analysis was used to divide household wealth into five equal categories based on quintiles (poorer, poor, middle, rich, and richer). The lowest two categories were merged as “poor” and the two highest categories were merged as “rich”. Place of delivery had 14 categories but recoded into two categories: delivered at home (own home or others home), and delivered at health facility (private or public health facility). Possession of vaccination card was coded in to five categories, and recoded into two: “Yes”- those who had vaccination cards and they were seen at interview; “No”- those who did not have cards or they were not seen during interview. Marital status was originally coded in to six categories and then recoded into two categories: currently married (those who are currently married and those living together), currently not married (those never married and those formerly married). Exposure to media was recoded from exposure to TV, radio, and news paper or magazine and coded in to two categories as “ever exposed” for those who ever exposed to both media and “Not at all” for those who never exposed to either media. Postnatal check-up, ANC visit, place of residence, region and all other variables considered in this analysis were coded as it was provided in Table 1.

**Table 1:** Distribution and crude association of full immunization and selected characteristics of respondents (weighted *n* = 1338)

Variable	Weighted No. (Weighted %)	Full Immunization		OR(95% CI)	*P*-value**
		No (%)	Yes (%)		
Maternal Education					
No education	597(44.6)	152(25.4)	445(74.6)	1	
Primary	298(22.3)	33(11.0)	265(89.0)	2.75(1.74–4.37)	< 0.001
Middle or above	443(33.1)	43(9.7)	400(90.3)	3.16(2.09–4.78)	< 0.001
Household characteristics					
Place of residence					
Urban	428(32.0)	44(10.3)	385(89.8)	1	
Rural	910(68.0)	184(20.2)	726(79.8)	0.45(0.30–0.66)	< 0.001
Wealth index					
Poor	536(41.1)	143(26.8)	392(73.2)	1	
Middle	292(22.2)	42 (14.3)	250(85.8)	2.20(1.43–3.37)	< 0.001
Rich	510(36.6)	43(8.4)	468(91.6)	3.75(2.48–5.66)	< 0.001
Region					
DKB	20(1.5)	8(34.5)	13(65.5)	1	
SKB	162(12.1)	39(24.1)	122(75.9)	1.66(0.87–3.14)	0.122
Gash-Bar	331(24.7)	78(23.5)	253(76.5)	1.72(0.96–3.07)	0.07
Anseba	206(15.4)	35(17.1)	171(82.9)	2.55(1.31–4.96)	0.006
Debub	382(28.5)	52(13.7)	330(86.3)	3.31(1.88–5.82)	< 0.001
Maekel	237(17.8)	16(6.9)	221(93.1)	7.14(3.51–14.53)	< 0.001
Mothers characteristics					
Age group					
15–24	335(25.0)	62(18.5)	273(81.5)	1	
25–29	385(28.8)	57(14.8)	328(85.2)	1.3(0.83–2.04)	0.247
30–34	289(21.6)	56(19.5)	233(80.5)	0.94(0.58–1.52)	0.796
35–39	229(17.1)	35(15.1)	194(84.9)	1.27(0.76–2.15)	0.361
40–49	100(7.5)	18(17.6)	83(82.4)	1.07(0.59–1.94)	0.833
Mothers’ Occupation					
Currently working	1053(79.8)	182(17.2)	872(82.8)	1	
Currently not working	267(20.2)	44(16.6)	223(83.4)	1.05(0.72–1.53)	0.81
Marital status					
Currently not married	84(6.3)	15(18.1)	68(81.9)	1	
Currently married	1237(93.7)	21,117.0)	1026(83.0)	1.07(0.56–2.04)	0.826
Exposure to mass-media					
Not at all	858(64.2)	163(19.0)	695(81.0)	1	
VEver exposed	480(35.8)	64(13.4)	415(86.6)	1.52(1.08–2.15)	0.018
Mother’s characteristics	Weighted No. (Weighted %)	Full Immunization		OR(95%CI)	*P*-value**
		No (%)	Yes (%)		
Religion					
Christian	780(58.6)	98(12.5)	682(87.5)	1	< 0.001
Muslim	551(41.4)	129(23.4)	423(76.6)	0.47(0.33–0.66)	
ANC visit					
No visit	112(8.4)	42(37.4)	70(62.7)	1	
1–3	433(32.4)	74(17.1)	359(82.9)	2.89(1.59–5.26)	0.001
4 or above	793(59.3)	112(14.4)	681(85.6)	3.63(2.08–6.34)	< 0.001
Postnatal check-up					
No	1061(79.4)	189(17.8)	872(82.2)	1	
Yes	275(20.6)	39(14.3)	236(85.8)	1.3(0.88–1.91)	0.188
Distance to health facility					
Big problem	495(37.5)	105(21.2)	390(78.8)	1	
Not big problem	826(62.5)	122(14.8)	703(85.2)	1.55(1.11–2.16)	0.01
Fathers characteristics					
Father’s Education					
No education	456(35.2)	124(27.2)	332(72.8)	1	
Primary	249(19.2)	35(13.9)	215(86.1)	2.32(1.44–3.75)	0.001
Middle	244(18.8)	26(10.7)	218(89.3)	3.13(1.87–5.23)	< 0.001
Secondary or above	347(26.8)	38(11.6)	308(88.4)	2.91(1.90–4.45)	< 0.001
Father’s Occupation					
Did not work	30(3.0)	5(16.4)	25(83.6)	1	
Professional/technical/ managerial..	96(7.3)	8(8.1)	88(91.9)	2.22(0.65–7.59)	0.201
Clerical/sales/services	603(47.0)	81(13.4)	522(86.6)	1.26(0.48–3.31)	0.632
Manual	181(13.8)	30(16.6)	151(83.4)	0.99(0.36–2.69)	0.978
Agriculture	370(29.0)	98(26.5)	272(73.5)	0.54(0.21–1.40	0.207
Child characteristics					
Vaccination card					
No	199(14.9)	115(57.5)	85(42.5)	1	
Yes	1139(85.1)	113(9.9)	1026(90.1)	12.29(8.43–17.91)	< 0.001
Sex					
Male	699(52.3)	121(17.3)	579(82.7)	1	
Female	638(47.7)	107(16.7)	532(83.3)	1.04(0.77–1.40)	0.81
Birth order					
First born	262(19.6)	39(14.9)	223(85.1)	1	
2–3	512(38.2)	77(15.0)	434(85.0)	0.99(0.62–1.59)	0.973
4–5	307(22.9)	57(18.8)	249(81.3)	0.76(0.46–1.26)	0.29
6+	258(19.3)	54(21.1)	203(78.9)	0.65(0.40–1.07)	0.092
Place of delivery					
Home	514(38.4)	166(20.1)	658(79.9)	1	
vHealth facility	824(61.6)	62(12.1)	452(87.9)	1.83(1.27–2.64)	0.001

NB. difference in number is due to missing values **p-value from Wald test

### Statistical analysis

Data were tabulated to understand the distribution of each variable and also to check for missing values, and data sparsity. There were missing data (65) for some variables including marital status [14], maternal occupation [15], antenatal visit (53), fathers’ education (36) and fathers’ occupation (50). Univariable analysis was done based on all observations; however, records with missing data were excluded from multivariable analysis. Since the missing values were relatively small (5% of the selected sub-population), excluding them from the analysis was unlikely to introduce bias. Variables chosen to be included in the final model were checked for collinearity by cross tabulating each other as well as by comparing the odds ratio and standard error, to check stability of the estimates, using the model with and without the variable.

To determined potential confounders, each of those chosen variables was cross tabulated with maternal education as well as with full immunization; hence, variables which were associated with both maternal education and full immunization were identified. Those covariates which were associated with both, exposure and outcome of interest were assessed to learn preliminary evidence for potential confounders using logistic regression by single adjustment for each variable. Besides, an interaction test was done to test whether each variable modified the association using adjusted Wald test. Those variables that changed the odds ratio by 10% or more to the crude odds ratio of the association between maternal education and full immunization were considered as confounders. Furthermore, each variable was assessed whether it was an independent risk factor of full immunization using logistic regression. In the final model, multivariable logistic regression, covariates which were either confounders or independent risk factors were considered. An adjusted Wald test was used to test whether a variable improved the model fit. Thus the final model was built based on factors that improved the fit of the model. Adjusted Wald test was also implemented to test whether an interaction existed between maternal education and wealth index, and between maternal education and region.

Data analysis was conducted using Stata v14 software. Since, this study was complex cluster sampling the sample was not self-weighted, hence a weighting variable was included during analysis to take account of clustering effect using svyset command. Though the sample size (unweighted or the actual number) considered in this study was 1323, the weighted number rose to 1338. Descriptive analysis was done and results were summarized as frequencies and percentages for categorical variables to report background characteristics of the household, mother, father and the child. Thus, all results including but not limited to frequencies, percentages, and odds ratio were weighted. Univariate analysis was done using logistic regression that accounted for survey design to produce odds ratio (OR) and 95% confidence interval (CI). *P*-values of the crude analysis were obtained from the Wald test. Moreover, multiple logistic regression was carried out to produce odds ratio of the association of maternal education and immunization status by controlling the effect of other factors.

### Ethical consideration

The EPHS 2010 survey was conducted by the Eritrea National Statistics Office (NSO) in collaboration with Ministry of Health (MOH) of the state of Eritrea. The interviewers read an informed consent to each respondent (or parent/guardian when children involved) before each interview or biomarker test was conducted; hence, it was only after an informed consent was sought that the interview was conducted. The NSO granted the dataset following a formal request by the Policy, Planning and Human Resources Development department of the Ministry of Health.

## Results

### Socio-demographic characteristics of respondents

A total of 10,805 eligible women were identified; of those eligible women 10,243 (94.8%) women, in all selected households, were interviewed,however, 3.6% were not found at home, 1.1% incapacitated, and 0.5% refused to participate or withdrew. Of those interviewed mothers there were 1323 mothers of children aged 12–23 months, who were interviewed during the survey, representing 1338 mothers after weighting. The distribution of respondents by selected characteristic was given in Table 1. Forty-five percent of mothers had no education whereas 33% of mothers attained above primary level. The highest proportion (29%) of mothers were aged 25–29 years followed by women aged 15–24 (25%) and women aged 30–34 (22%). Majority (94%) of mothers were currently married. Eighty percent of respondents were unemployed during the 12 months prior to the survey. Six out of ten mothers (59%) were Christians. Merely 36% of mothers had ever exposed to mass media. Ninety-two percent of mothers had visited antenatal care at least once during their pregnancy where 60% visited 4 or more times. Almost four out of five (79%) of mothers didn’t attend postnatal care. Majority (63%) of mothers stated that distance to health facility was not a big problem. One third (35%) of fathers had no education and 27% of fathers attained secondary or higher level. Most of the fathers (47%) were employed as clerical or sales or services followed by employee of agriculture (29%). More than half (52%) of children were males. The highest proportion (38%) of children was second to third birth order followed by fourth to fifth birth order (23%). Eighty-five percent of children had vaccination cards and 62% of children were born at home.

### Vaccination coverage

**Fig. 1:**
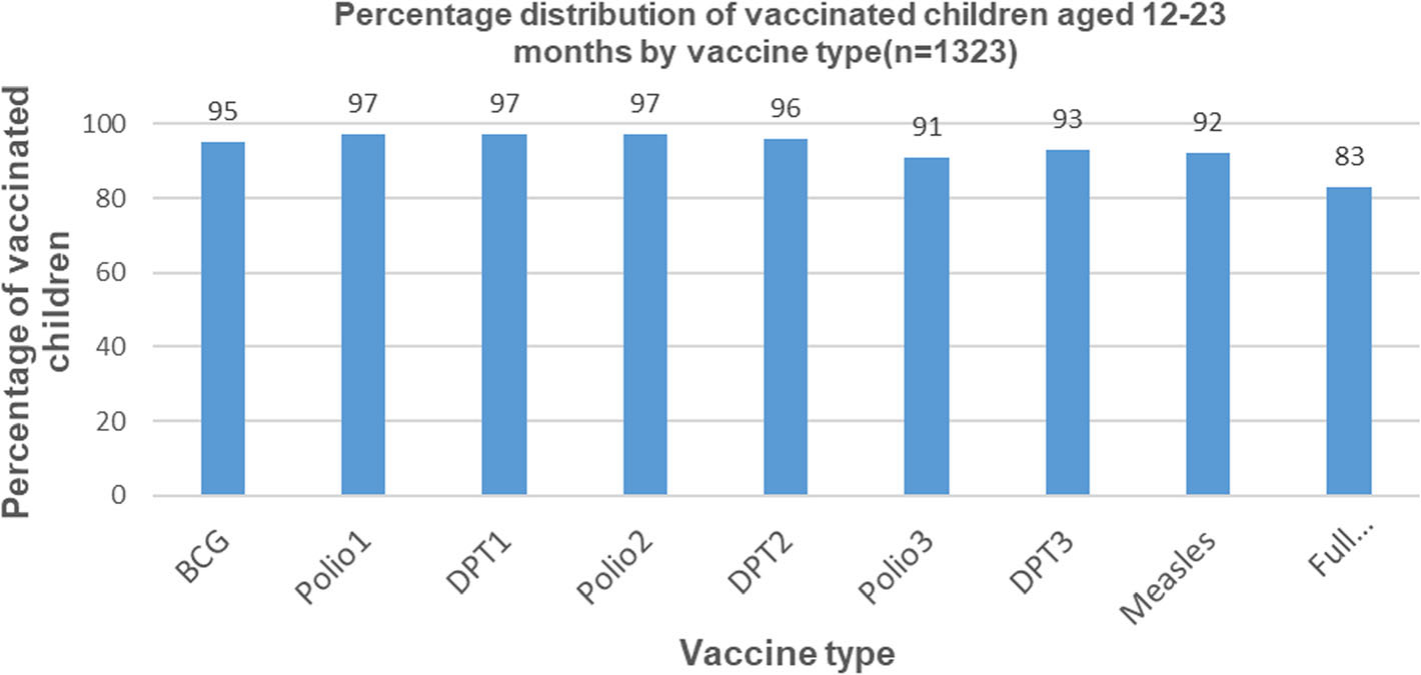
Percentage distribution of vaccinated children aged 12–23 months by vaccine type (*n* = 1323)

Vaccination coverage was above 90% across the vaccination continuum, as shown in Fig. 1. The drop out of children from DPT1 to DPT3 was 4%, and the drop out from polio 1 to polio 3 was 6%. Overall, 83% of children received all eight recommended vaccines, fully immunized. However, 17% of children either didn’t receive any vaccine at all or were partially vaccinated: 2% didn’t receive vaccine at all, and 15% were partially vaccinated.

### Bivariate analysis

The crude association between maternal education and immunization status and the association of immunization status and other variables was given in Table 1. The crude odds ratio indicated that there was a strong association between maternal education and full immunization. Children of mothers with primary level of education were 2.8 times and children of mothers with middle or above were 3.2 times more likely to be fully vaccinated than children of mothers with no education. Children of households with middle wealth level (OR = 2.2, 95% CI 1.4–3.4), rich households (OR = 3.8, 95% CI 2.5–5.7), residents of Maekel (OR = 7.1, 95% CI 3.5–14.5), Debub (OR = 3.3, 95% CI 1.9–5.8), and Anseba (OR = 2.6, 95%CI 1.3–5.0), were more likely to be fully immunized. On the other hand children of households resided in rural areas were less likely to be fully immunized (OR = 0.5, 95% CI 0.3–0.7). Furthermore, children of mothers who ever exposed to mass media (OR = 1.5, 95% CI 1.1–2.2), reported distance to health facility was not big problem (OR = 1.6, 95% CI 1.1–2.2), visited ANC 1–3 times (OR = 2.9, 95% CI1.6–5.3), visited 4 or more times (OR = 3.6, 95% CI 2.1–6.3) were fully immunized. Conversely, children of Muslim mothers were less likely to be fully vaccinated (OR = 0.5, 95% CI 0.3–0.7). Besides, children of fathers with primary (OR = 2.3, 95% CI 1.4–3.8), middle (OR = 3.1, 95% CI 1.9–5.2), and secondary or above (OR = 2.9, 95% CI 1.9–4.5) were fully immunized. Moreover, children born at health facility (OR = 1.8, 95% CI 1.3–2.6), and children who had a vaccination card (OR = 12.3, 95% CI 8.4–17.9) were more likely to be fully immunized.

The variables that were associated with maternal education were maternal age(*p* < 0.001), maternal occupation(*p* = 0.0001), marital status(*p* = 0.0004), exposure to mass media(*p* < 0.001), religion (*p* < 0.001), birth order(*p* < 0.001), vaccination card(*p* = 0.0446), fathers occupation(*p* < 0.001), fathers education(*p* < 0.001), wealth index(*p* < 0.001), residence(urban/rural)(*p* < 0.001),region(*p* < 0.001), ANC visit (*p* < 0.001),place of delivery(*p* < 0.001), postnatal check(*p* < 0.001), distance to health facility(*p* < 0.001). However, sex of a child (*p* = 0.6698) was not associated with maternal education.

### Multivariable analysis

Wealth index and region were identified as confounders of the relationship between maternal education and full immunization, whereas vaccination card was an independent risk factor. Thus, they were included in the final model. The estimates from the final models were presented in Table 2. Maternal ANC visit could be a mediating factor since educated mother could seek health services which could increase full immunization. Thus, two models were built to examine overall effect of maternal education on full immunization: excluding the potential mediating variable, ANC visit and including this variable. The effect of maternal education on full immunization adjusted for wealth index, region and possession of vaccination card without ANC visit was presented with Model1 and with ANC visit included was presented with Model2. So results of Model 1 indicated that the odds of full immunization among children of mothers with primary level still existed though the odds ratio reduced whereas the odd of full immunization among children of mother with middle or above level was attenuated. After adjusting for ANC, Model 2 showed that the impact of maternal education on full immunization among primary level still existed, though the odds ratio was reduced. But ANC visit only explained a small amount of the effect of maternal education on immunisation. Children of mothers with primary level education were more likely to be fully immunized than children of mother with no education, but the effect for mothers with middle or higher education disappeared. So, based on the results of the final model (model 2), the relationship between maternal education and full immunization was influenced by wealth index of household, mothers ANC visit, region and possession of a vaccination card. Children of mothers who attended ANC 1–3 times, children of rich households, children who had a vaccination card, and children who lived in Debub region were more likely to be fully immunized (Table 2). Using the final model (Model2) an interaction test between maternal education and both wealth index and region were examined with adjusted Wald test; however, neither wealth index (*p* = 0.1875) nor region (*p* = 0.7650) modified the association between maternal education and full immunization. Furthermore, the interaction test was done with Model1 as well and it was insignificant (data not shown).

**Table 2:** Final model of multivariable logistic regression, impact of maternal education on full immunization

Factor	Model 1^a^		Model 2^b^	
	OR(95% CI)	*p*-value	OR(95% CI)	*p*-value
Maternal Education				
No education	1		1	
Primary	2.51(1.40–4.49)	0.002	2.34(1.30–4.21)	0.005
Middle or above	1.60(0.91–2.78)	0.099	1.49(0.84–2.65)	0.173
Wealth index				
Poor	1		1	
Middle	1.51(0.92–2.47)	0.103	1.48(0.87–2.5)	0.148
Rich	2.53(1.48–4.33)	0.001	2.50(1.40–4.47)	0.002
Region				
DKB	1		1	
Maekel	1.24(0.96–5.23)	0.061	2.08(0.88–4.92)	0.096
SKB	1.42(0.68–2.99)	0.352	1.26(0.59–2.70)	0.549
Anseba	1.98(0.87–4.47)	0.102	1.63(0.70–3.77)	0.253
Gash-Barka	1.87(0.89–3.96)	0.100	1.60(0.74–3.46)	0.228
Debub	2.71(1.32–5.58)	< 0.007	2.47(1.18–5.18)	0.016
Vaccination card				
No	1		1	
Yes	12.73(8.38–19.35)	< 0.001	12.79(8.38–19.52)	< 0.001
ANC Visit				
No visit			1	
1–3			2.17(1.07–4.40)	0.031
4 and above			1.67(0.83–3.38)	0.151

^a^Model1 OR adjusted for wealth index, region and possession of vaccination card

^b^Modle2 OR adjusted for wealth index, region, possession of vaccination card and ANC visit

## Discussion

This study assessed the relationship between maternal education and full immunization among children aged 12–23 months in Eritrea using PHS data. Full vaccination coverage among children aged 12–23 months was 83%. In unadjusted analyses, children of mothers with primary, and middle or above education were more likely to be fully immunised. After adjusting for wealth, region and vaccination card ownership, the effect for primary education still existed, but for middle or above level of education was attenuated. The relationship between maternal education and full immunization was influenced by wealth index of household, mothers ANC visit, region and possession of a vaccination card. Children of mothers who attended ANC 1–3 times, children of rich households, children who had a vaccination card, and children who lived in Debub region were more likely to be fully immunized.

In multivariable analysis, the association of maternal education was assessed with two models to examine the role of ANC visit, ownership of vaccination card, region and wealth index. ANC visit could be a mediator; as higher maternal education could lead to higher use of health services. However, the magnitude of the effect of maternal education on immunisation was not greatly altered by adding ANC visit to the model, suggesting that it was not responsible for much of the effect of maternal education on immunisation. Possession of vaccination card was an independent risk factor and region was a confounder. The difference in vaccination status in the regions could be due to the fact that some regions like Gash Barka, Anseba and Semenawi Keih Bahri have nomadic people and hard to reach areas. But, in Debub region the people are static and access to health services is relatively good. However, it is not clear why Maekel region has lower vaccination status than Debub region despite that Maekel region has better access to health service. Wealth index could be a confounder or mediator since higher maternal education could lead to higher socio-economic status which leads to higher immunization. Conversely, wealth index could also independently have associated with maternal education and immunization since higher socio-economic status could lead to higher maternal education and immunization. Thus, with both models, including and excluding ANC visit, the association of maternal education and immunization status still existed, though the odds ratio was reduced. Hence, children of mothers with primary level or higher were more likely to be fully immunized than children of mother with no education.

An educated mother is expected to gather health information from health workers or mass media and this helps her to make better decisions and improve health seeking behaviour. A study conducted in rural Nigeria found that mother’s knowledge of immunization was significantly correlated with full immunization [16]. Other studies also found that maternal education was a determinant of immunization [8, 11, 14, 15, 17–20]. However, several other studies didn’t find evidence of association between maternal education and immunization [14, 17, 21, 22]. Therefore, the present study supports the hypothesis that children of educated mother are more likely to be fully immunized compared to children of uneducated mothers.

The strength of this study was the use of nationally representative data, large sample, and high response rate (95%). Hence, the result could be generalized to the whole nation. Furthermore, we explored the potential influence of a lot of covariates in order to control their effect on the association between maternal education and full immunization. However, the study had several limitations. The information was obtained from records on vaccination cards or mothers recall of vaccination; hence, there might be misclassification error. The full immunization could have been overestimated since mothers could have reported children as vaccinated when in fact they were not vaccinated (i.e. social desirability bias). The information of the outcome as well as the primary exposure and other determinants were collected simultaneously, though it was very likely that maternal education occurred before immunization. The finding of this study suggested a relationship between maternal education and immunization uptake. However, there could be uncontrolled confounding due to other factors such as maternal tetanus toxoid vaccination, ethnicity vaccine availability and health workers(staff) commitment and absence that could explain the determinants of full immunization were not assessed. The immunization status and other independent variables included in this study refer to the situations 8 years back which do not reflect the current situation in the country. Thus, a further rigorous study that addresses these mentioned limitations should be conducted. Moreover, a qualitative study should also be included to assess why mothers with no education are less likely to have their children fully vaccinated.

The implication of this study is to investigate the impact of maternal education on immunization and the role of other factors on the immunization in order to recommend strategies that improve immunization among children in Eritrea. Strategies should be developed that target uneducated mothers who do not attend ANC, from poor households and living in regions Anseba, Maekel, Gash-Barka, and Semenawi Keih Bahri with educational messages and community engagement activities. In the longer term, improving the educational opportunities and outcomes for women will lead to better maternal and child health in general, because educated mothers will make better decisions on health seeking behaviour, including immunization, antenatal care, postnatal care, and possession of a vaccination card.

## Conclusion

This study investigated the relationship between maternal education and full immunization, among children age 12–23 months in Eritrea, using data from PHS 2010. This study found that children of mothers who attained primary level were more likely to be fully vaccinated than children of mothers with no education. The relationship between maternal education and full immunization was influenced by wealth index of household, mothers ANC visit, region and possession of a vaccination card. Children of mothers who attended ANC 1–3 times, children of rich households, children who had a vaccination card, and children who lived in Debub region were more likely to be fully immunized. Strategies to enhance immunization, such as health education and information campaign, keeping vaccination card, and outreach services, should be planned that target mothers who are: uneducated, do not attend ANC, from poor households and living in the regions of Anseba, Maekel, Gash-Barka, and Semenawi Keih Bahri. Thus, this study found an association between maternal education and full immunization among children aged 12–23 months in Eritrea.

## Data Availability

The data that support the findings of this study are available from [NSO] but restrictions apply to the availability of these data, which were used under license for the current study, and so are not publicly available. Data are however available from the authors upon reasonable request and with permission of the National Statistics Office.

## Abbreviations

ANC: Antenatal Care
BCG: Bacillus Calmette-Guérin
DKB: Debubawi Keih Bahri
DPT: Diphtheria, Pertussis, Tetanus
EPHS: Eritrea Population and Health Survey
EPI: Extended Program of Immunization
ERI-CMYP: Eritrea Comprehensive Mid-Year Plan
GAVI: Global Alliance for Vaccine and Immunization
GVAP: Global Vaccine Action Plan
MOH: Ministry of Health
NSO: National Statistics Office
PHS: Population and Health Survey
SKB: Semenawi Keih Bahri
UNICEF: United Nations Children’s Fund
WHO: World Health Organization
